# Acquired Dermal Macular Hyperpigmentation Mimicking Dowling Degos Disease: A Case Report

**DOI:** 10.7759/cureus.28150

**Published:** 2022-08-18

**Authors:** Walaa A Ahmed, Sara B Badirah, Rahaf A Abdulwahab, Khalid Al Hawsawi

**Affiliations:** 1 Medicine and Surgery, Umm AlQura University, Makkah, SAU; 2 Medical Student, Umm AlQura University, Makkah, SAU; 3 Dermatology, King Abdulaziz Hospital, Makkah, SAU

**Keywords:** hyperpigmentary disorder, reticulate pigmentary disorder, dowling degos disease, acquired dermal macular hyperpigmentation, lichen planus pigmentosus

## Abstract

Acquired dermal macular hyperpigmentation (ADMH) is a recently coined term to encompass lichen planus pigmentosus (LPP), erythema dyschromicum perstans (EDP), and Riehl’s melanosis. Here we report a 60 -year- old female, with an insignificant past medical history, who presented to the dermatology clinic, with slightly itchy skin lesions on her body. The lesions were slowly increasing in number over the last 10 years. The patient was otherwise healthy and was not taking any medications. A review of systems was unremarkable. There was no similar case in the family and the parents did not show consanguinity. Skin examination revealed multiple well-defined non-scaly brownish macules scattered on her body. In addition, bilateral macules and papules were present in the inframammary folds. There were no skin lesions in the axillae, groin, and intergluteal folds. Differential diagnoses include Dowling Degos Disease (DDD), LPP, and EDP. A 4 mm punch skin biopsy was taken from skin lesions under the breast. It revealed hyperkeratosis, hypergranulosis, and acanthosis. The dermis showed a band-like infiltrate of mononuclear histiocytic cellular infiltrate with basal layer degeneration. According to the above clinicopathological findings, the diagnosis of lichen planus was made. The patient was reassured. She was started on hydroxychlorquine 200 mg tab bid, a topical steroid, and topical calcineurin inhibitors, and was asked to follow up regularly in the dermatology clinic.

## Introduction

Acquired dermal macular hyperpigmentation (ADMH) is a term coined recently for acquired dermatoses that are characterized by acquired dermal macular hyperpigmentation. It includes lichen planus pigmentosus (LPP), erythema dyschromicum perstans (EDP), and Riehl's melanosis (pigmented contact dermatitis). These conditions are similar in both clinical and histopathological features [1]. They are clinically characterized by the appearance of gray-brown to blue-gray macules and patches. Histologically, there is a lichenoid tissue reaction present [2].

Dowling-Degos disease (DDD) is a rare autosomal dominant inherited skin disorder characterized by reticulated hyperpigmentation and sometimes lentigo-like macules [3]. It typically begins during the third to the fourth decade of life. The pigmentation typically first appears in the axillae and groin and can later spread to other skin folds, neck, trunk, and inner aspects of the arms and thighs [3]. Some patients report pruritus of the affected flexural areas. We report a case of ADMH that mimic DDD.

## Case presentation

**Figure 1:**
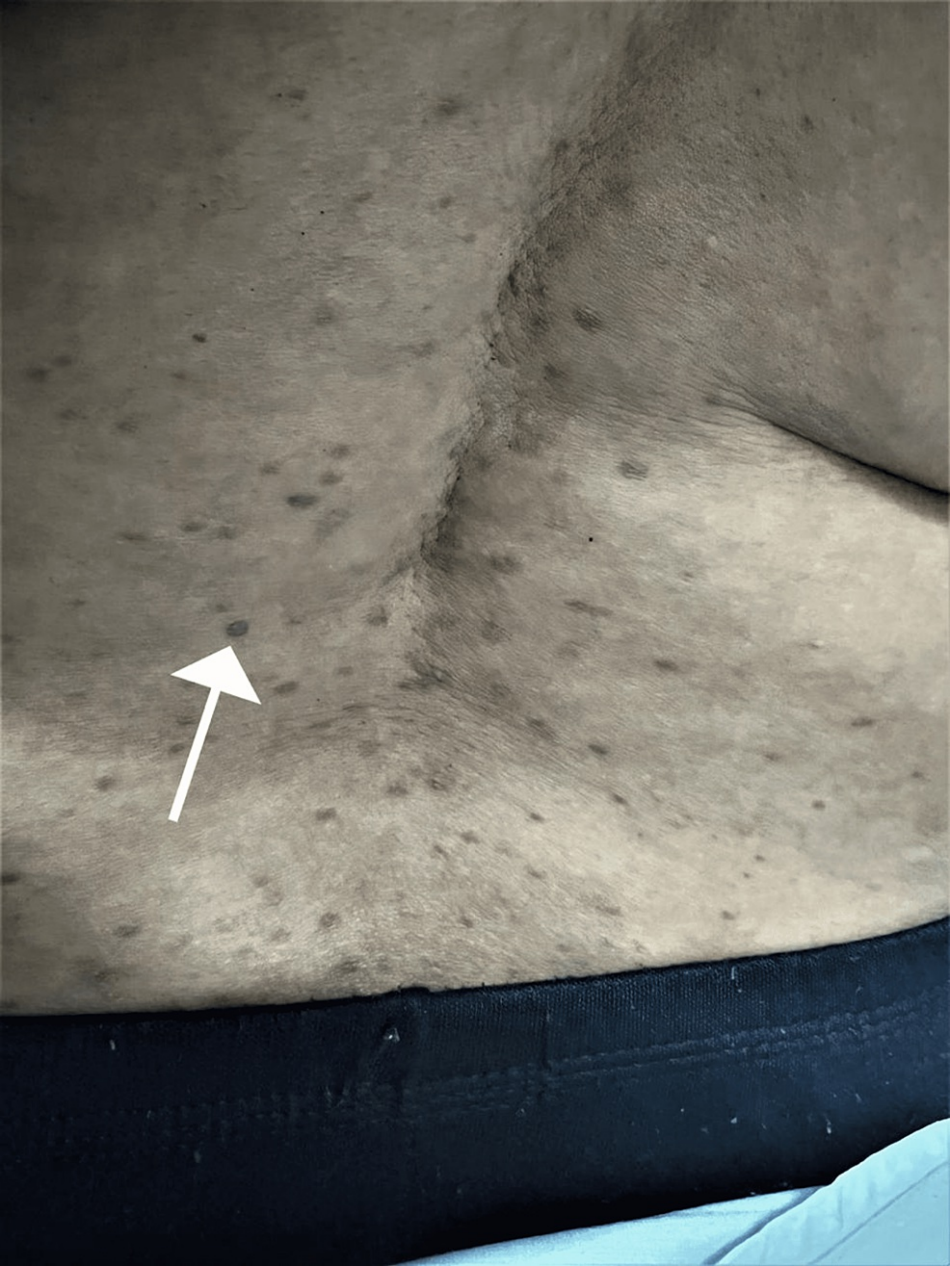
The back showing multiple non-scaly brownish macules.

**Figure 2:**
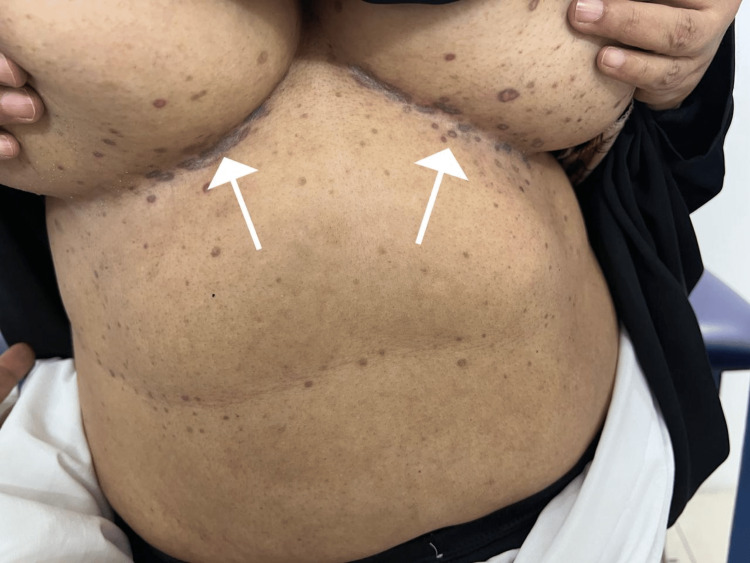
There were macules and papules under her breast bilaterally.

**Figure 3:**
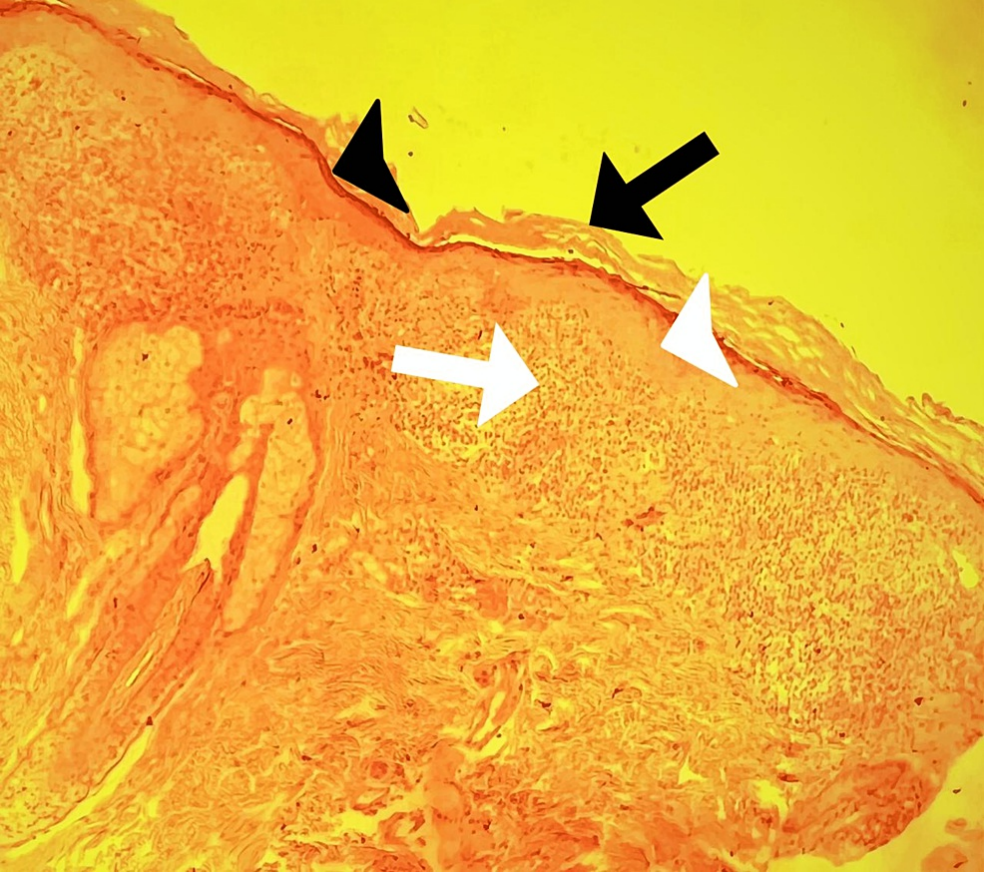
Punch skin biopsy, the epidermis showed hyperkeratosis, hypergranulosis, and acanthosis. The dermis showed a band-like infiltrate of mononuclear histiocytic cellular infiltrate with basal layer degeneration. (hematoxylin& eosin stain; original magnification, x20)  Black arrow: hyperkeratosis  Black arrowhead: hypergranulosis  White arrowhead: acanthosis  White arrow: band-like infiltrate

 A 60-year-old female, with an insignificant past medical history, presented to the dermatology clinic, with slightly itchy skin lesions on her body. The lesions were slowly increasing in number over the last 10 years. The patient was otherwise healthy and was not taking any medications. A review of systems was unremarkable. There was no similar case in the family and the parents did not show consanguinity. Skin examination revealed multiple well-defined non-scaly brownish macules scattered on her body, some macules look scaly (Figure 1). There were also bilateral macules and papules in the inframammary folds (Figure 2). There were no skin lesions in the axillae, groin, and intergluteal folds. Differential diagnoses included DDD, LPP, and EDP. A 4 mm punch skin biopsy was taken from skin lesions under the breast. It revealed hyperkeratosis, hypergranulosis, and acanthosis. The dermis showed a band-like infiltrate of mononuclear histiocytic cellular infiltrate with basal layer degeneration (Figure 3). According to the above clinicopathological findings, the diagnosis of lichen planus was made. She was started on hydroxychloroquine 200 mg tab bid, a topical steroid, and topical calcineurin inhibitors, and was followed up regularly in the dermatology clinic. The patient has not shown up until the time of this report. 

## Discussion

ADMH is a recently coined term to encompass LPP, EDP, and Riehl’s melanosis. The clinically and histopathologically similarity between these three entities and the existence of overlapping features in some cases have led experts to unifying these diseases under the term ADMH [1]. Clinically, they are characterized by asymptomatic gray-brown macules and patches [2]. LPP favors sun-exposed areas and flexures, whereas the lesions of EDP are usually present in sun-protected areas. Riehl’s melanosis favors sites on which contactants such as fragrance and other ingredients of cosmetic products have been applied [1,3]. Histopathologically they are characterized by evidence of current or resolved interface dermatitis including vacuolar degeneration of the basal layer, lichenoid tissue reaction, pigment incontinence, and melanophages [2].

DDD is a rare genodermatosis that is inherited as autosomal dominant. Sporadic cases have been reported. It appears as reticulated hyper-pigmented macules and/or papules that initially appear in the axillae and groin followed by the intergluteal and inframammary folds, neck, and trunk. Comedone-like lesions on the back or neck, pitted perioral scars, epidermoid cysts, and hidradenitis suppurativa are additional features in some patients [2,4,5]. However, our patient had none of these. The slow onset of skin lesions in our patient in the fourth decade of life and their spread from inframammary areas to other parts of her body led to an initial diagnosis of DDD. However, the histopathological features were typical for lichen planus. Histopathologically, DDD is characterized by elongated branched (antler-like) rete ridges with increased melanin pigmentation at the tips of the epidermis. 

The treatment is difficult. Topical treatment includes steroids, calcineurin inhibitors, and retinoids. Systemic treatment includes steroids, retinoids including acitretin and isotretinoin, hydroxychloroquine, methotrexate, azathioprine, and phototherapy [4,5].

## Conclusions

ADMH is a recently coined term to include LPP, EDP, and Riehl’s melanosis. Sometimes ADMH and DDD look clinically similar, as in our case, However, these clinical entities can be distinguished by their characteristic histological features. This case report aims to raise the awareness of this condition. We recommend further studies with a higher level of evidence to investigate and assess this condition.
